# Non-vitamin K Oral Anticoagulants and Anti-seizure Medications: A Retrospective Cohort Study

**DOI:** 10.3389/fneur.2020.588053

**Published:** 2021-02-26

**Authors:** Chen-Jui Ho, Shih-Hsuan Chen, Chih-Hsiang Lin, Yan-Ting Lu, Che-Wei Hsu, Meng-Han Tsai

**Affiliations:** ^1^Department of Neurology, College of Medicine, Kaohsiung Chang Gung Memorial Hospital, Chang Gung University, Kaohsiung, Taiwan; ^2^School of Medicine, College of Medicine, Chang Gung University, Taoyuan, Taiwan

**Keywords:** epilepsy, ASM, drug–drug interaction, stroke, NOAC

## Abstract

**Purpose:** Concerns of drug–drug interactions (DDIs) between anti-seizure medications (ASMs) and non-vitamin K oral anticoagulants (NOACs) have emerged in recent case reports and guidelines. Theoretically, the induction of hepatic cytochrome P450 3A4 (CYP3A4) enzyme and permeability glycoprotein (P-GP) efflux transporter protein systems may reduce the effect of NOACs. We aimed to investigate whether such DDIs are clinically relevant in a real-world situation.

**Methods:** We retrospectively reviewed 320 ischemic stroke patients with atrial fibrillation (Af) and grouped them according to different potential interactions with CYP3A4 and P-GP. Ischemic stroke events, transient ischemic attack (TIA) events, follow-up duration, baseline characteristics, concomitant ASMs, and stroke risk factors were collected. Statistical analysis included Kaplan–Meier survival curves and the log-rank test.

**Results:** Overall, 320 ischemic stroke with Af patients received NOACs. Among the NOAC users, 75 also took ASMs, including 56 that have potential DDIs: 43 (13.4%) were categorized as potential CYP and P-GP DDIs and 13 (4.1%) as P-GP-only DDIs. The remaining 264 (82.5%) patients were used as controls including 19 exposed to nonsignificant DDI ASMs and 245 patients without ASM exposure. The incidence rates of recurrent stroke/TIA events in both CYP3A4 and P-GP DDIs, P-GP DDIs only, and no DDIs were 7.5, 2.1, and 8.4/100 person-years, respectively. Kaplan–Meier survival curves and the log-rank test did not show significant differences among the groups.

**Conclusions:** The recurrent stroke rate of NOAC users with potential DDIs was not higher than in those without potential DDIs in this single-institute study. Our results suggest that theoretical interactions between ASMs and NOACs may not be as severe as previously thought in a real-world situation.

## Introduction

Atrial fibrillation (Af) is a major risk factor for cardioembolic stroke and requires oral anticoagulant (OAC) therapy. Post-stroke epilepsy has been reported to occur in 5–17% of ischemic stroke patients, which is even higher (31%) in patients with cardioembolic stroke (1). Long-term anti-seizure medication (ASM) treatment is necessary for seizure control in these patients. Recently, non-vitamin K oral anticoagulants (NOACs) have become the mainstream treatment for non-valvular Af patients due to their efficacy and safety profile (2). Real-world studies have reported lower incidence rates of stroke with better adherence to guidelines (3, 4). With the increasing use of NOACs, the issue of potential drug–drug interactions (DDIs) in polypharmacy was raised in the 2018 European Heart Rhythm Association Practical Guide on the use of NOACs in patients with Af, including the possible interactions between ASMs and NOACs (5).

Theoretically, DDIs between NOACs and ASMs mainly focus on the effect of permeability glycoprotein (P-GP) and cytochrome P450 3A4 enzyme (CYP3A4) systems (5). Induction of CYP3A4 may increase hepatic clearance of drugs, and induction of P-GP may increase the re-secretion of medications in the gut and clearance in the kidneys (6, 7). Many ASMs are known to affect these systems. Oxcarbazepine and topiramate can induce CYP3A4 enzyme (8, 9), while levetiracetam can induce P-GP (10). In addition, carbamazepine, phenobarbital, phenytoin, and valproic acid have been shown to be able to induce both CYP3A4 and P-GP, although the effect varies in different models (11–18). Critical DDIs may be associated with failure of the anticoagulation effect and expose the patients to the risk of recurrent stroke and systemic embolism (19, 20). On the other hand, switching ASMs to avoid DDIs has been associated with a 16.7–21.7% increased risk in 6-month seizure recurrence rate (21, 22). Therefore, selecting the most appropriate ASMs for patients with post-stroke epilepsy and Af needs to consider the DDIs between ASMs and NOACs. Currently, the guideline recommendations for DDIs are mainly based on cell/animal models or case reports (8, 10). Due to the lack of strong clinical evidence, this study aimed to investigate whether theoretical DDIs between ASMs and NOACs have an impact on stroke prevention in a real-world setting.

## Materials and Methods

### Study Design

We retrospectively reviewed patients admitted for ischemic stroke due to Af at the Department of Neurology of Kaohsiung Chang Gung Memorial Hospital from January 2013 to December 2017. This study was approved by the local Institutional Review Board of Kaohsiung Chang Gung Memorial Hospital (IRB No. 201901169B0).

### Definitions and Criteria

Ischemic stroke was diagnosed by an episode of neurologic dysfunction caused by vascular stenosis or occlusion leading to focal cerebral, spinal cord, or retinal infarction within a specific vascular territory. Cardioembolic stroke was defined as arterial occlusion presumably due to an embolus arising in the heart (23). Transient ischemic attack (TIA) was diagnosed by neurologic symptoms of ischemic origin that lasted for least than 24 h (24).

### Clinical Assessment

We included ischemic stroke patients aged ≥18 years who used NOACs for Af and had at least 3 months of follow-up data. Patients who received other medications that potentially interact with CYP3A4 or P-GP (either induction or inhibition) were excluded: verapamil, amiodarone, quinidine, human immunodeficiency virus (HIV) medications, rifampicin, ketoconazole, cyclosporine, and tacrolimus (5, 25, 26).

The patients' age, sex, underlying medical condition, follow-up duration, stroke risk factors, and medication history were recorded. The NOAC regimen was evaluated according to the 2014 American Heart Association (AHA)/American College of Cardiology (ACC)/Heart Rhythm Society (HRS) guidelines for Af management (2). The primary endpoint was any ischemic stroke or TIA event during follow-up.

**Figure 1:**
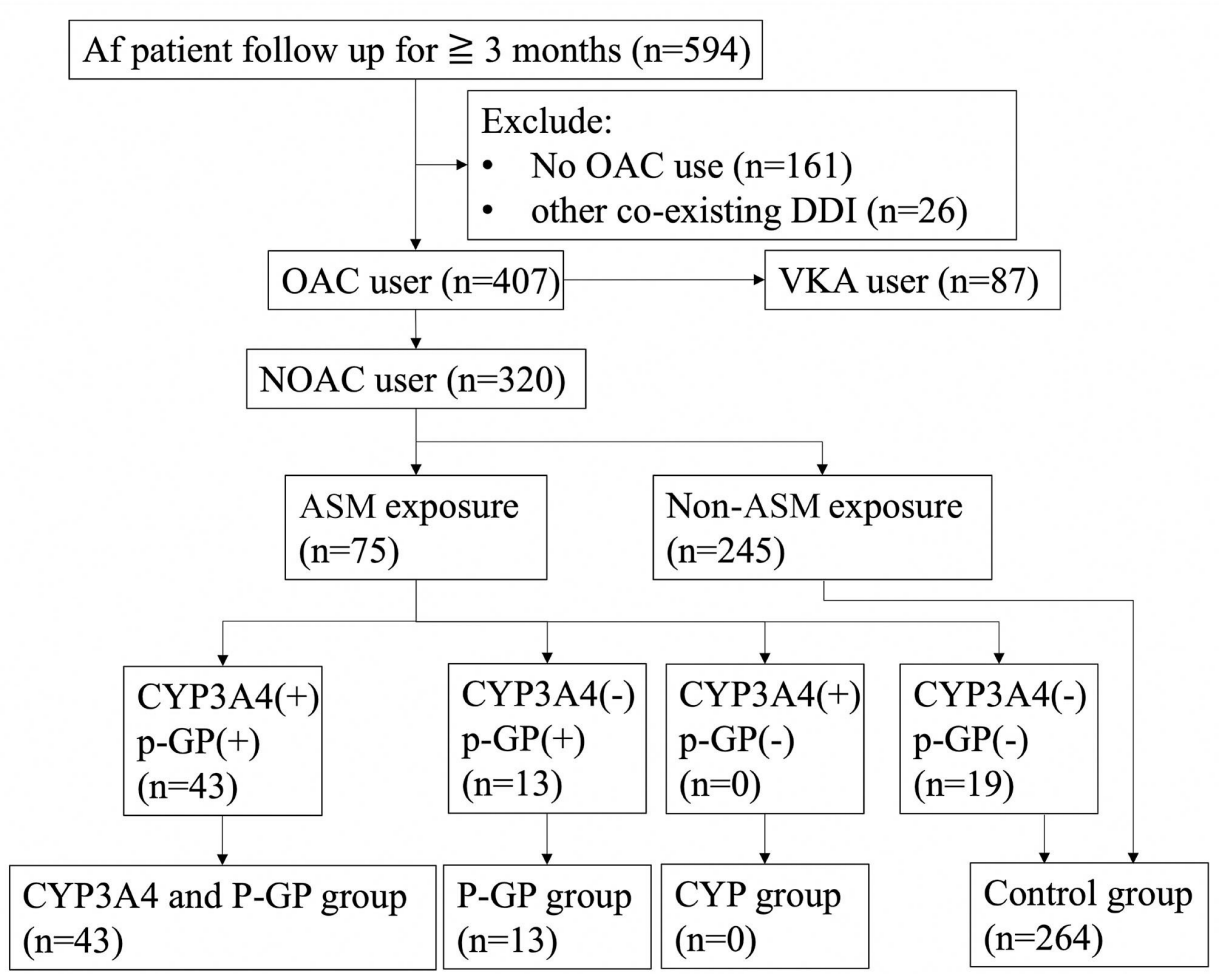
Flow diagramof patient inclusion and exclusion. Af, atrial fibrillation; OAC, oral anticoagulant; VKA, vitamin K antagonist; DDI, drug–drug interaction; ASM, anti-seizure medication; CYP3A4, cytochrome P450 3A4; P-GP, permeability glycoprotein; *n*, number.

Carbamazepine, oxcarbazepine, phenobarbital, phenytoin, topiramate, and valproic acid were defined as being CYP3A4 inducers, and levetiracetam, carbamazepine, phenobarbital, phenytoin, and valproic acid were defined as being P-GP inducers. The patients were divided into groups according to the ASMs as: “both CYP3A4 and P-GP inducers,” “CYP3A4 inducers,” “P-GP inducers,” and “control group” (Figure 1). The control group was made up of patients without ASM exposure and patients receiving ASMs that have no interaction with CYP3A4 and P-GP.

Because ASMs may have varying effects on CYP3A4, we further divided the ASMs into strong CYP3A4 inducer group (carbamazepine, phenobarbital, phenytoin) and weak CYP3A4 inducer group (topiramate, valproic acid, oxcarbazepine) (8, 12, 15, 18, 27).

For patient follow-up, patients were followed up regularly in our clinic every 1–3 months according to their clinical condition. Only the regular follow-up periods in our clinic were included for analysis. As for the ASM co-administered groups, only the times when NOAC and ASMs were both given were counted as the duration of follow-up.

### Statistical Analysis

All statistical analyses were performed using SPSS v.24.0 for Mac (SPSS Inc., Chicago, IL, USA). Descriptive summaries were reported as the mean ± standard deviation for continuous variables and as number (percentage) for categorical variables. For nonparametric variables, we used the median value with the interquartile range (IQR) and the Mann–Whitney U test with two-sided test. Incidence rates were calculated as the number of ischemic events divided by the time at risk and presented per 100 person-years. The cumulative incidence of ischemic events was evaluated using Kaplan–Meier survival curves, which were visually inspected to check the proportional hazards assumption. Differences between groups were evaluated using the log-rank test. A *p* value of ≤ 0.05 was considered to be statistically significant.

## Results

During the study period, 594 patients with a diagnosis of Af and ischemic stroke were reviewed. Twenty-six patients were excluded because they received other co-medications with potential CYP3A4 or P-GP interaction besides ASMs. Eighty-seven patients receiving vitamin K antagonist (VKA) and another 161 patients who did not receive OAC treatment were excluded.

A total of 320 stroke with Af patients received NOAC treatment, including 75 patients who received ASMs. Among them, 56 patients were exposed to CYP3A4 or P-GP inducing ASMs and 19 exposed to ASMs without significant DDIs. Forty-three of them (13.4%) were classified into the CYP3A4 plus P-GP group and 13 (4.1%) were classified as P-GP-only group. The remaining 264 (82.5%) were used as controls, including 19 patients with nonsignificant DDI ASM exposure and 245 patients who did not take ASMs. No patient was classified as CYP3A4-only group. No significant differences were noted between the CYP3A4 and P-GP group and control group except that the CYP3A4 and P-GP group had significantly fewer male patients and dyslipidemia compared with the control group. Similarly, no significant differences were noted between the P-GP group and control group except that the P-GP group had significantly fewer male patients and a longer follow-up duration than those of the control group (Table 1).

**Table 1:** The DDIs of ASMs in patients taking NOACs.

**Total N**	**CYP and P-GP**	**P-GP**	**Control**	***p* value**	**P-GP vs. Control**	**CYP and P-GP vs. Control**
	**43**	**13**	**264**	**CYP and P-GP vs. P-GP**		
Age (year, median, IQR)	71 (67.3–79.3)	72.2 (68–77.1)	75.3 (69–81.2)	0.741	0.147	0.179
Male sex (*N*, %)	18 (41.9)	4 (30.8)	164 (60)	0.477	**0.031**	**0.019**
Follow-up (months, median, IQR)	12.3 (4.8–28.8)	30 (15.6–38.4)	20.4 (10.8–40.8)	0.003	**0.027**	0.067
CHA2DS2-VASc (median, IQR)	3 (2-4)	3 (3-4)	3 (2-4)	0.364	0.669	0.272
CHF (*N*, %)	7 (16.3)	42 (15.9)	2 (15.4)	0.939	0.96	0.951
Hypertension (*N*, %)	19 (44.2)	152 (57.6)	6 (46.2)	0.901	0.418	0.102
Vascular disease history (*N*, %)	6 (14)	35 (13.3)	2 (15.4)	0.898	0.826	0.901
DM (*N*, %)	6 (14)	65 (24.6)	3 (23.1)	0.437	0.9	0.125
Prior stroke/TIA history (*N*, %)	3 (7)	34 (12.9)	2 (15.4)	0.356	0.793	0.271
Creatinine (mg/dl) (median, IQR)	0.8 (0.77–0.89)	0.86 (0.72–1.11)	0.96 (0.77–1.14)	0.467	0.053	0.241
Dyslipidemia (*N*, %)	10 (23.3)	5 (38.5)	125 (47.3)	0.282	0.532	**0.003**
BMI (median, IQR)	25.8 (24.7–28.5)	27.2 (25–28.8)	24 (21.3–27.2)	0.069	0.091	0.82
Smoking (*N*, %)	7 (16.3)	1 (7.7)	50 (18.9)	0.442	0.308	0.678
Nonstandard dose reduction (*N*, %)	26 (72.9)	10 (76.9)	164 (64.1)	0.282	0.282	0.836
New-onset heart failure (*N*, %)	1 (2.3)	0	3 (1.1)			0.454
Mortality (*N*, %)	8 (18)	2 (15)	32 (12)	1	0.665	0.23
Recurrent admission (100 p-yr, *N*)	61 (11)	35 (3)	50 (81)	0.705	0.584	0.295
Embolic event IR (100 p-yr, *N*)	7.3 (2)	0 (0)	7.4 (42)			0.809
Hemorrhagic event IR (100 p-yr, *N*)	2.9 (2)	5.4 (2)	3.8 (22)	0.628	0.708	0.843
Total event IR (100 p-yr, *N*)	10.2 (7)	5.4 (2)	11.2 (64)	0.486	0.374	0.662

*NOAC, non-vitamin K oral anticoagulant; CYP, cytochrome P450 3A4; P-GP, permeability glycoprotein; TIA, transient ischemic attack; CHF, congestive heart failure; DM, diabetes mellitus; IQR, interquartile range; N, number; %, percentage; 100 p-yr, 100 persons per year; BMI, body mass index; DDI, drug–drug interaction; ASM, anti-seizure medication. Bold values indicate statistically significant*.

Among patients taking ASMs with NOACs (*N* = 56), the average number of ASMs used was 1.4 (range 1–5). Thirty-seven used one ASM, 16 used two ASMs, two used three ASMs, and one used five ASMs. All patients in the P-GP group used one ASM (*N* = 13), which was levetiracetam. For patients in the CYP and P-GP group, they used levetiracetam, valproic acid, phenytoin, topiramate, lamotrigine, carbamazepine, or zonisamide (Table 2).

**Table 2:** ASM doses among groups.

		**CYP and P-GP group**	**P-GP group**	**Control group**
	Total *N*	43	13	264
ASM daily dose (median, *N*, IQR)	LEV	1,000 [15 (35%), 1,000–1,975]	1, 000 [13 (100%), 1,000–1,600]	
	VPA	1,300 [26 (60%), 1,000–1,500]		
	PHT	300 [17 (40%), 300–300]		
	LMT	275 [3 (7%), 212.5–337.5]		
	TPM	600 [1 (2%)]		
	CBZ	800 [2 (5%), 800–800]		
	ZNS	500 [2 (5%), 500–500]		
	PER	2 [6 (14%), 2–4]	2 [1 (8%)]	2 [9 (3%), 2–4]
	LCS	300 [4 (9%), 200–400]	200 [2 (15%), 200–200]	200 [10 (4%), 200–200]

*ASM, anti-seizure medication; IQR, interquartile range; N, number; NOAC, non-vitamin K oral anticoagulant; CYP, cytochrome P450 3A4; P-GP, permeability glycoprotein; LEV, levetiracetam; VPA, valproic acid; PHT, phenytoin; TPM, topiramate; LMT, lamotrigine; CBZ, carbamazepine; ZNS, zonisamide; PER, perampanel; LCS, lacosamide*.

There was no significant difference in the incidence of recurrent stroke/TIA events between the CYP3A4 and P-GP group and the control group (7.3 vs. 7.4/100 person-years, *p* = 0.809). Although the P-GP group had no recurrent stroke/TIA events, it also had the smallest number of patients (*n* = 20). During the follow-up period, no systemic embolic events were observed. Among them, five (12%) of the CYP3A4 and P-GP group, two (15%) of the P-GP group, and 43 (16%) of the controls were lost to follow-up due to failed to return to the clinics or moved to other institutes for follow-up.

Because the effect of CYP3A4 may be varied between ASMs, we further divided the CYP3A4 inducer category into strong and weak groups. The incidence of ischemic events for the strong CYP3A4 inducer group is 3.3 (100 person-years, total/event *N* = 17/1), weak CYP3A4 inducer group is 10.6 (100 person-years, total/event *N* = 26/4), and control group is 7.4 (100 person-years, total/event *N* = 264/42). No statistically significant difference was observed between groups (Table 3).

**Table 3:** DDIs of strong and weak CYP3A4 inducers on NOAC user.

	**Strong inducer**	**Weak Inducer**	**Control**	***p* value**
**ASM**	**Carbamazepine phenobarbital phenytoin**	**Topiramate valproic acid oxcarbazepine**		**Strong vs. control**	**Weak vs. control**	**Strong vs. weak**
Total *N*	17	26	264			
Age (median, IQR)	77.6 (66–78.7)	72 (67–83)	75.3 (69–81.2)	0.388	0.362	0.886
Male Gender (*N*, %)	3 (17.6)	16 (62)	164 (60)	**<0.001**	0.834	**0.003**
Follow-up (months, median, IQR)	20.4 (8.4–32.4)	13.2 (4.8–26.4)	20.4 (10.8–40.8)	0.45	**0.041**	0.583
CHA2DS2-VASc (median, IQR)	4 (2.25–4.75)	3 (2-3)	3 (2-4)	0.68	0.112	0.22
Dyslipidemia (*N*, %)	5 (29.4)	7 (27)	125 (47.3)	0.108	0.057	0.951
Smoking (*N*, %)	1 (5.9)	5 (19)	50 (18.9)	0.154	0.961	0.199
Creatinine (mg/dl, median, IQR)	0.84 (0.65–0.95)	0.95 (0.78–1.38)	0.96 (0.77–1.14)	**0.014**	0.593	0.068
Nonstandard dose reduction (*N*, %)	10 (58.8)	18 (69.2)	164 (64.1)	0.58	0.846	0.359
Embolic event IR (*N*) (100 p-yr)	3.3 (1)	10.6 (4)	7.4 (42)	0.477	0.344	0.512

*NOAC, non-vitamin K oral anticoagulant; CYP, cytochrome P450 3A4; IQR, interquartile range; N, number; %, percentage; CHF, chronic heart failure; DM, diabetes mellitus; TIA, transient ischemic attack; IR, incidence rate; 100 p-yr, 100 persons per year; DDI, drug–drug interaction. Bold values indicate statistically significant*.

Because an insufficient dose of NOACs may affect the risk of recurrent stroke, we further analyzed this confounding factor. Nonstandard dose reductions were noted in 200 (62.5%) patients. The incidence rates of standard dose and nonstandard dose reductions were 6.2 (100 person-years) and 7.3 (100 person-years) (*p* = 0.815), respectively. We performed a subanalysis of the patients who received an adequate NOAC dose and NOAC underdose groups. For adequate NOAC dose, there was no significant difference in the incidence rate among the CYP3A4 and P-GP, P-GP, and control groups [4.1, 0, and 6.7 per 100 person-years (*p* = 0.941); event-free ratio, 88.4, 100, and 84.1%, respectively]. Similarly, for NOAC underdose group, no significant difference in the incidence rate among the CYP3A4 and P-GP, P-GP, and control groups [9.3, 0, and 7.7 per 100 person-years (*p* = 0.802); event-free ratio, 84.6, 100, and 83.5%, respectively].

**Figure 2:**
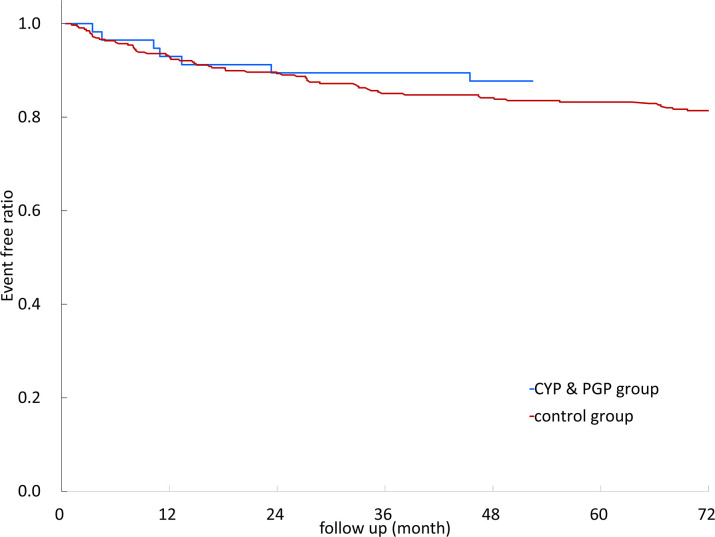
Kaplan–Meier survival curves of non-vitamin K oral anticoagulants (NOACs) with different anti-seizure medication (ASM) drug–drug interactions. CYP3A4, cytochrome P450 3A4; P-GP, permeability glycoprotein; CYP3A4&P-GP, CYP3A4 and P-GP.

Kaplan–Meier survival curves of recurrent stroke/TIA events with different DDIs are illustrated in Figure 2. The results showed no significant difference between different DDIs and control groups (log-rank test, *p* = 0.809).

## Discussion

In this retrospective cohort study, we reported real-world data on DDIs between ASMs and NOACs. Surprisingly, we found that the use of CYP3A4 and/or P-GP inducers did not significantly alter the incidence of recurrent stroke/TIA even in patients who received underdosed NOACs despite the limited cohort size collected from a single institute.

Previous studies have suggested that several ASMs may affect the CYP3A4 and P-GP system. Carbamazepine and phenytoin could induce CYP3A4 and P-GP in healthy volunteers; however, the potency and efficacy were low compared with strong inducers such as rifampin (11, 12). Phenobarbital and valproic acid have also been reported to induce CYP3A4 and P-GP based on mouse/rat models and human cell line studies (13–18). NOACs are mainly metabolized by CYP3A4 and secreted into the gut by the P-GP system. With the increasing use of NOACs, DDIs have become an important issue, especially as elderly patients often have multiple medical conditions. The 2018 European Heart Rhythm Association Practical Guide reviewed potential DDIs for NOACs and highlighted that carbamazepine, levetiracetam, oxcarbazepine, phenobarbital, phenytoin, topiramate, and valproic acid should not be used or used with caution or avoided in patients taking NOACs (5). Although theoretical DDIs have been observed in animal and cell line models, this may not always reflect the same degree of interaction in humans. This is demonstrated in our study where we did not observe an increase in cardioembolic stroke in patients receiving potential interacting ASMs, both in the standard NOACs dose or underdose groups.

There are several possible explanations for this discrepancy between theoretical interactions and real-world data. First, the dose of ASMs may be relevant. For example, the enzyme-inducing effect is known to occur at a higher dose of many ASMs (≥1,200 mg/day for oxcarbazepine, ≥800 mg/day for carbamazepine, and ≥400 mg/day for topiramate) (9, 28). Patients in our study usually received lower ASM doses that may not induce liver enzymes. Second, the P-GP system is known to have a species-specific effect so that it can be induced by phenytoin and levetiracetam in mice but not in humans (29, 30). The phase 1 study of levetiracetam on healthy volunteers did not have significant DDIs on P-GP (29).

In this retrospective study, we found that co-medication of potential interacting ASMs to NOACs did not increase the risk of recurrent ischemic events in post-stroke epilepsy patients. This suggests that DDIs involving CYP3A4 and/or P-GP in ASMs may not be a major clinical concern. However, this finding should be interpreted with caution. The study sample size was relatively small (*N* = 320), included only Asian population, and retrospective with limited follow-up duration [median 20.2 (IQR: 13.2–30) months], or lost to follow-up, which may underestimate the risk of recurrent stroke. In addition, the high prevalence of underdosing NOACs in this study group may affect the outcomes. The use of lower-dose NOAC in the Asian population is relatively common (15–59%) because of the concerns of higher bleeding tendencies to VKA and lower body mass (31, 32). Underdosing of NOACs may exacerbate the risk of DDIs on recurrent stroke due to a lower blood concentration; however, we did not observe a significant increase in embolic events in the NOAC underdose group. We also did not observe a significant increase of embolic stroke when comparing patients with underdosed NOAC vs. standard NOAC dose. This is in accordance with the Outcomes Registry for Better Informed Treatment of Atrial Fibrillation II study (ORBIT-AF II) where there is no difference between standard and underdosing groups (33). Another limitation was that direct NOAC serum concentration monitoring was not routinely performed in our clinical practice. Lastly, similar to warfarin, it is possible that genetic factors that influence the metabolism or transportation of the NOACs may have an effect on the DDIs, which was not evaluated in this study.

In conclusion, our single-institute study suggests that DDIs between ASMs and NOACs in Asian post-stroke epilepsy patients may not be as severe as previously thought in a single institutional real-world situation. Further international studies with a larger sample size, different ethnicities, direct monitoring of NOAC serum concentration, and longer follow-up duration or meta-analysis are warranted to clarify the clinical significance of such DDIs.

## Data Availability Statement

The datasets presented in this article are not readily available because the dataset generated and analyzed during the current study is not publicly available. The consent form participants did not cover data sharing but are available from the corresponding author on reasonable request. Requests to access the datasets should be directed to menghan@cgmh.org.tw.

## Ethics Statement

The studies involving human participants were reviewed and approved by Chang Gung Medical Foundation Institutional Reviewer Board 201901169B0. Written informed consent for participation was not required for this study in accordance with the national legislation and the institutional requirements.

## Author Contributions

C-JH, S-HC, C-HL, Y-TL, C-WH, and M-HT contributed to the acquisition and interpretation of the data and revising the manuscript for intellectual content. C-JH, S-HC, and M-HT contributed to the design and conceptualization of the study; analysis and interpretation of the data, drafting, revising, and final approval of the manuscript for intellectual content. All authors contributed to the article and approved the submitted version.

## Conflict of Interest

The authors declare that the research was conducted in the absence of any commercial or financial relationships that could be construed as a potential conflict of interest.
